# Next Generation Sequencing of Acute Myeloid Leukemia: Influencing Prognosis

**DOI:** 10.1186/1471-2164-16-S1-S5

**Published:** 2015-01-15

**Authors:** Asad Muhammad Ilyas, Sultan Ahmad, Muhammad Faheem, Muhammad Imran Naseer, Taha A Kumosani, Muhammad Hussain Al-Qahtani, Mamdooh Gari, Farid Ahmed

**Affiliations:** 1 Department of Biochemistry, King Abdulaziz University, Jeddah, KSA; 2 King Fahd Medical Research Center, King Abdulaziz University, Jeddah, KSA; 3 Centre of Excellence in Genomic Medicine Research, King Abdulaziz University, Jeddah, KSA; 4 KACST Technology Innovation Center in Personalized Medicine, King Abdulaziz University, Jeddah, Kingdom of Saudi Arabia

**Keywords:** Acute myeloid leukemia, Next generation sequencing, Exome sequencing, RNA-Seq, AML prognosis, Targeted AML therapy

## Abstract

Acute myeloid leukemia (AML) is a clonal disorder of the blood forming cells characterized by accumulation of immature blast cells in the bone marrow and peripheral blood. Being a heterogeneous disease, AML has been the subject of numerous studies that focus on unraveling the clinical, cellular and molecular variations with the aim to better understand and treat the disease. Cytogenetic-risk stratification of AML is well established and commonly used by clinicians in therapeutic management of cases with chromosomal abnormalities. Successive inclusion of novel molecular abnormalities has substantially modified the classification and understanding of AML in the past decade. With the advent of next generation sequencing (NGS) technologies the discovery of novel molecular abnormalities has accelerated. NGS has been successfully used in several studies and has provided an unprecedented overview of molecular aberrations as well as the underlying clonal evolution in AML. The extended spectrum of abnormalities discovered by NGS is currently under extensive validation for their prognostic and therapeutic values. In this review we highlight the recent advances in the understanding of AML in the NGS era.

## Introduction

Acute myeloid leukemia (AML) is the second most common hematological tumor and is characterized by increased proliferation and impaired maturation of early myeloid cells, leading to an accumulation of immature blood cells. The disease is now recognized as a very heterogeneous entity. Studies of chromosomal abnormalities by cytogenetics, followed by molecular genetics approach have played a significant role in deciphering the heterogeneity of AML as well as providing a profound insight into the biology of leukemia. Recurrent chromosomal and molecular defects detected at diagnosis have provided valuable prognostic information such as: response to induction chemotherapy, relapse risk and overall survival (OS). The 2008 WHO classification characterized AML based on recurrent genetic abnormalities and mutations in two oncogenes (*NPM1* and *CEBPA*) [1]. Based on these genetic alterations, this classification system divides patients into three categories: favorable, intermediate and unfavorable. The cytogenetic profile for patients with favorable prognosis includes *PML-RARA*, *RUNX1-RUNX1T* and *CBFB-MYH11* translocations. Acute promyelocytic leukemia (APL) showing *PML-RARA* are treated on ATRA- and anthracycline-based or ATRA- and arsenic trioxide-based protocols, whereas the core binding factor (CBF) leukemias with *RUNX1-RUNX1T* and *CBFB-MYH11* are treated with intensive chemotherapy involving cytarabine and are characterized by relatively favorable prognosis [2]. Cytogenetic abnormalities associated with unfavorable prognosis include monosomies of chromosomes 5 or 7, 11q23 alterations (excluding t(9;11)), inv(3), t(6;9), monosomal karyotype (defined as having greater or equal to two autosomal monosomies or a single monosomy with additional structural abnormalities) and complex karyotype (presence of three or more chromosomal abnormalities). These patients require allogeneic bone marrow transplantation during their first remission. About 50% of AML patients having cytogenetically normal karyotype (CN-AML) are categorized in the intermediate group [3]. Multiple mutations emerge in this group, which have prognostic effect determining the outcome for the therapy. The most common mutation is *NPM1* in CN-AML. Patients having *NPM1* mutation in the absence of a FLT3-ITD mutation have better remission rate and overall survival rate. On the other hand, CN-AML patients positive for *FLT3-*internal tandem duplication (*FLT3-*ITD) have poor prognosis [1,3]. Morphological, immunological as well as cytogenetic evaluation of peripheral blood and bone marrow have been the mainstay of leukemia diagnosis. Recently updated recommendations of the European Leukemia Net (ELN) for the diagnosis and management of AML has been reviewed extensively by Dohner H *et al*[4].

The advent of next generation sequencing (NGS) technologies has expedited the discovery of novel genetic lesions in AML. A number of re-sequencing studies in AML patients, particularly those showing normal cytogenetics, have led to the discovery of several new mutations (including *DNMT3A*, *IDH1*, *IDH2*, *TET2*) [5]. An effort to use the newly identified mutations, alone or in conjunction with previously characterized genetic anomalies, for gaining prognostic insights has begun. The therapy of AML has largely remained unchanged from the standard 3+7 regimen, for this reason the most important advances to current prognostic markers will be identifying those gene mutation(s) that can prospectively stratify patients who will benefit from an allogeneic hematopoietic stem cell transplant (HSCT). However, given the risks associated with HSCT, the identification of underlying genomic alterations in AML has a great potential for the incorporation of rationally-targeted therapies, generating a hope for improvement in treatment outcome. In this review, we have outlined the recent advances in the genomics of AML namely: the use of different NGS methodologies, novel mutations identified using NGS and their clinical impact.

## Next Generation Sequencing Technologies

In the past decade, conventional Sanger sequencing method has been overtaken by several new technological advances collectively known as next generation sequencing (NGS). These technologies apply different target enrichment strategies and clonal amplification of the DNA resulting in the possibility of sequencing millions of DNA strands in parallel. This massively parallel sequencing facilitates high-throughput sequencing with a significant reduction in costs. The advent of NGS has significantly accelerated the effort to understand the molecular basis of cancers [6]. Recent development of “bench-top NGS Instruments” such as Ion Torrent PGM from Life Technologies and Mi-Seq from Illumina has been of tremendous utility in clinical settings and individual laboratories. Ion torrent PGM is based on semiconductor sequencing in which detection is done on a semiconductor chip. This technology detects the pH change due to release of hydrogen ions when a new nucleotide is inserted during synthesis [7]. Mi-Seq technology adopted the ‘sequencing by synthesis’ approach in which the template amplification and data analysis is combined in a single instrument. These instruments are smaller in size, having high throughput, high accuracy and less running time. In addition, these machines provide shorter read lengths which makes them more suitable for clinical and diagnostic applications [8]. Comprehensive reviews are available that discuss various technological aspects of NGS and its recent advancements [8-10].

## AML Whole genome sequencing

Whole genome sequencing (WGS) based on NGS techniques offers the possibility to identify the complete range of genomic alterations: point mutations, indels, copy number changes, and structural rearrangements including translocations, cryptic rearrangements, inversions and complex rearrangements in the whole genome. The first whole cancer genome to be sequenced using NGS was from an AML patient in 2008 by Ley *et al *[11]. Genomic DNA samples from tumor and skin tissues of the cytogenetically normal AML patient were sequenced. The group performed 32.7-fold ‘haploid’ coverage (98 billion bases) for the tumor and 13.9-fold coverage (41.8 billion bases) for the normal sample, resulting in identification of 10 somatic mutations. Out of these, two recurrent somatic mutations (*FLT3* and *NPM1*) have well-defined implications in AML. The other eight were somatic non-synonymous novel mutations. A year later, another AML patient with normal cytogenetics was sequenced by the same group. They identified sixty four somatic mutations which include mutations in the coding sequences of genes and in the conserved regulatory portions of the genome [12]. The identified mutations were validated in a larger cohort of 187 AML samples with recurring mutations found in *NPM1*, *NRAS* and *IDH1* genes. In a 2010 study, Ley *et al* used the paired end deep sequencing approach to re-sequence the AML samples from their first study. This led to the identification of a recurrent somatic mutation in the DNA methyltransferase-3-alpha gene (*DNMT3A*). The frequency of *DNMT3A* mutation was 22% (62/281). They concluded that *DNMT3A* mutation is a recurrent mutation in patients with de novo AML classified into intermediate risk category [13]. Welch *et al* used whole genome sequencing to accurately diagnose an AML case that was referred to their institute for allogeneic stem cell transplantation. The patient had complex cytogenetic profile associated with unfavorable prognosis, no detection of *PML-RARA* fusion transcript by fluorescence in situ hybridization (FISH) but appeared to have APL. Whole genome sequencing was performed that led to the identification of *PML-RARA* fusion gene and two other fusion genes: *LOXL1-PML* and *RARA-LOXL1*. Finally, the patient was treated as APL with ATRA consolidation leading to extended remission [14]. This was an example showing how NGS based diagnosis could be useful in guiding clinicians to select the treatment path. A recent comprehensive study by the Cancer Genome Atlas (TCGA) Research Network analyzed whole genomes of 50 de novo AML cases. This study also combined whole exome and methylome analysis, and concluded that AML genomes have fewer mutations than other cancers, with an average of 13 gene mutations of which an average of 5 genes show recurrent mutations in AML [15].

In another study using whole genome sequencing, Welch *et al* compared the genomes of 12 AML M3 cases having known initiation event (*PML-RAR*) vs genomes of 12 AML M1 CN-AML patients, to identify the initiating mutation in CN-AML [16]. It was found that both AML groups have approximately same number of overall mutations, however, AML M1 genomes have mutations in *NPM1*, *DNMT3A* and *IDH1* but not in AML M3. It was concluded that these mutations might act as major initiating mutations in this group. *FLT3-*ITD mutation was found in both genomes suggesting it to be a cooperating mutation rather than an initiating mutation. Although 13 recurrently mutated genes were found only in AML M1 samples, there were 9 recurrent mutations detected in both groups suggesting cooperation of initiating mutations can lead to AML. This study also proposed that most of the mutations in the AML genome are random events or exits as background mutations in the hematopoietic stem cell. After the initiating mutation event, cells expand with the same history of mutations to become the founding clone. This clone can acquire additional mutations to evolve into sub clones that can contribute to disease progression or relapse. Later on, Ding *et al* reported the clonal evolution of mutations in acute and relapsed myeloid leukemia that have been reviewed extensively elsewhere [17-19].

## Transcriptome sequencing

Transcriptome sequencing is a technique that can sequence transcribed genes, coding RNA and non-coding RNA (ncRNA) that represents the complete transcriptome. The main advantage of transcriptome sequencing over WGS is that it provides: gene expression level information, identification of expressed fusion transcripts, post-transcriptional modification including alternative splicing, single nucleotide variants (SNV) and RNA editing events [20]. RNA Sequencing (RNA-Seq) is a recent refinement that typically involve poly-A selection, cDNA synthesis by reverse transcription, fragmentation followed by ligation of sequencing primers [21]. Using paired-end RNA-Seq, Wen *et al* identified novel fusion transcript in a group of 45 AML patients that included 29 CN-AML cases, 8 cases with abnormal karyotype and 8 cases with no karyotype information [22]. The sensitivity of RNA-Seq method was shown by detection of well-known fusions in AML (*RUNX*-*RUNX1T1*, *MLL*-*MLLT1* and *MLL*-*MLLT3*) with abnormal karyotype. Of the 134 identified fusions identified in all AML cases, seven transcript fusions were found exclusively in CN-AML. Three *CIITA*-*DEXI* fusions on chromosome 16 were detected in 14 out of 29 CN-AML patients. However, this significant finding is yet to be reproduced by other studies. Recently, Masetti *et al* used transcriptome sequencing to identify two novel fusion transcripts in pediatric CN-AML: *CBFA2T3-GLIS2* fusion and *DHH-RHEBL1* fusion [23,24]. In a large cohort validation, *CBFA2T3-GLIS2* transcript was recurrently found in pediatric CN-AML cases and predicted poor outcome [23]. The *DHH-RHEBL1* transcript was detected in 40% of *CBFA2T3-GLIS2*-rearranged patients and conferred even worse prognosis than patients carrying *CBFA2T3-GLIS*-rearragment [24]. Although the role of fusion transcripts detected in this study remains to be elucidated in the context of leukemogenesis, this study has highlighted the possibility of using transcriptome sequencing for identification of chromosomal aberrations even in a large heterogeneous group of CN-AML patients, which are otherwise considered to be devoid of chromosomal rearrangements.

## Exome Sequencing

The exome is the coding region of the genome and accounts for only about 1% of the whole genome. Selectively sequencing the exome is an effective and economic strategy to identify novel sequence variations in the coding region of the genome [25]. With the recent development in high-throughput sequence capture methods, exome sequencing has become an attractive and practical approach for investigating coding region variations [26,27]. Several studies have been conducted to sequence the AML exomes and the trend is increasing. Using exome sequencing in nine AML-M5 patients, Yan *et al* identified mutations in 14 genes; out of which six genes having mutations previously implicated in cancers or other diseases are: *DNMT3A*, *NSD1*, *GATA2*, *CCND3*, *ATP2A2* and *C10orf2*. The key finding in this study was the presence of three different *DNMT3A* variants in 23 out of 112 samples (20.5%), with individuals carrying *DNMT3A* mutants showing inferior prognosis as compared to ones carrying wild type *DNMT3A.* In addition *MLL* abnormalities including *MLL* translocations, *NPM1* and *NRA*S mutations and *FLT3* internal tandem duplications were also identified [28]. Grief *et al* sequenced three *PML-RARA* positive APL patients in leukemic and remission states. APL specific somatic mutations were identified in *WT1*, *KRAS*, *HDX*, *and LYN* that contributes to the pathogenesis of the disease [29]. Using whole exome sequencing, Grossman *et al* studied CN-AML patients with no mutation in *NPM1*, *CEBPA*, *FLT3-ITD*, *IDH* and *MLL-PTD* genes (AML index patients). Mutations in 11 genes were detected of which the most likely candidates for AML pathogenesis were: *DNMT3A*, *BCOR*, *YY2* and *SSRP1*. The frequency of *BCOR* mutation was 17.1% in AML index patients, whereas the overall frequency of *BCOR* in unselected CN-AML was 3.8%. Mutations in *BCOR* were not detected in 131 AML cases having various cytogenetic abnormalities. Interestingly, 50% of the mutated *BCOR* patients have *DNMT3A* mutations suggesting that BCOR and DNMT3A might have role in the pathogenesis of CN-AML with wild type *FLT3* and *CEBPA *[30]. Using exome sequencing, Grief *et al* identified novel *GATA2* zinc finger 1 mutation in 2 of 5 biallelic *CEBPA* (bi*CEBPA*) AML patients. Further mutational screening in 33 bi*CEBPA* AML patients showed mutations in: *GATA2* (39.4%), *FLT3-*ITD (18.2%), *DNMT3A* (9.1%), *IKZF1* (6.1%). All mutations in *GATA2* were found in the highly conserved N-terminal zinc finger domain (ZF1 domain) [31]. Opatz *et al* identified a novel N676K mutation in fms-related tyrosine kinase 3 (*FLT3*) using exome sequencing in core binding factor (CBF) leukemia [32]. This study underscores the potential use of exome sequencing in detecting novel sequence alterations even in extensively studied genes.

## Targeted next generation gene sequencing

NGS techniques are continuously evolving to decrease the cost and time of sequencing, however, the amount of data generated and the effort in data analysis may not be manageable in a clinical diagnostic setting. On the other hand, a full molecular diagnostic profiling of AML may be increasingly required for clinical management of patients. For this reason, targeted gene sequencing method has gained popularity in recent years. This is a promising and effective way for targeted molecular genotyping of AML patients. Duncavage *et al* used targeted gene sequencing in AML cell lines to validate a method for identifying prognostically significant gene mutations including translocations, single nucleotide variants (SNVs) and indels on a single platform [33]. Conte *et al* described targeted DNA capture method using cRNA bait combined with deep sequencing for identification of 24 recurrently mutated genes in CN-AML [34]. They sequenced seven AML patients and identified 20 exonic mutations and 1 intronic mutation in known recurrently mutated genes like *FLT3*, *NPM1*, *CEBPA*, *DNMT3A*, *TET2*, *IDH1 *&* 2*, *WT1 *>and* KRAS*. Out of these 20 exonic mutations, 11 were single base substitutions and 9 were small indels. Two to four mutations were present in each AML patient [34]. In a recent study by Kihara *et al*, targeted NGS of 51 genes were performed. A total of 505 mutations in 44 genes were identified, out of which five genes: *FLT3*, *NPM1*, *CEBPA*, *DNMT3A *>and* KIT*, were mutated in more than 10% of the patients [35]. In addition, this comprehensive study analyzed the clinical features and prognostic impact of mutations and suggested alternative risk stratification of AML patients by inclusion of *DNMT3A*, *MLL-PTD* and *TP53* genes in the proposed European Leukemia Net (ELN) system [35,36]. With time, advancement in targeted gene sequencing may prove useful in clinical settings for the detection of known chromosomal translocations and gene mutations, requiring less procedural time and leading to correct diagnosis and risk stratification of AML patients.

## Expanding spectrum of molecular genetic abnormalities in AML: Implications for prognosis and therapy

Diverse cytogenetic and molecular genetic alterations in AML have profound influence on prognostic and therapeutic decisions taken by clinicians. Cytogenetic aberrations at diagnosis have become well defined genetic biomarkers for AML classification, treatment outcome and stand as independent prognostic indicators. Core-binding factor-acute myeloid leukemia (CBF-AML) defined by the presence of t(8;21) or inv(16), show good outcome with intensive chemotherapy when compared with other AML subtypes or CN-AML [37]. Complex karyotype AML (CK-AML) and AML with single autosomal monosomies have poor prognosis, however AML patients having multiple autosomal monosomies or autosomal monosomies in combination with at least one chromosomal structural abnormality have extremely poor outcome [38,39]. Further prognostic stratification of poor prognosis AML is possible with the inclusion of additional molecular alterations. For example, CK- AML with TP53 alterations had significantly lower complete remission (CR) rates and worse relapse-free survival (RFS), overall survival (OS), and event-free survival (EFS) as compared to those without TP53 alterations [40]. In CBF-AML, the presence of a *KIT* mutation has been shown to bring an unfavorable influence on the outcome [41]. CN-AML that lacks these cytogenetic profiles accounts for 40-50% of adult AML and is the most heterogeneous sub-group of AML. Recurrent molecular aberrations like mutations in *FLT3*, *NPM1*, *and CEBPA* have been useful in prognostic sub classification of CN-AML and led to the inclusion of these markers in the revised WHO classification of AML in 2008 [42]. *FLT3* internal tandem duplication (ITDs) are found in approximately 20% of all AML cases and between 28%-34% in CN-AML [43]. AML with *FLT3-*ITD show worse prognosis and is associated with shorter EFS, RFS and OS when treated with conventional chemotherapy as compared to the *FLT3-*ITD negative patients [3,43]. In contrast, patients having no *FLT3-ITD* but mutated bi*CEBPA* or *NPM1* gene show favorable outcome [44,45].

With the advent of NGS technologies, several new mutations have been described (table 1). Since then, a number of studies have been started with the aim to determine the mutations that contribute to disease pathogenesis and impact clinical outcomes. The following novel gene mutations (summarized in table 2) have been recently studied for their prognostic values either independently or with other known molecular abnormalities: *DNMT3A*, *IDH1*, *IDH2*, *TET2 *and *ASXL1*.

**Table 1:** Mutations identified in Acute Myeloid Leukemia by different approaches of Next Generation Sequencing

NGS Techniques	References	AML type	Recurrent Mutations identified (Frequency)	Mutation Type
Whole Genome Seq	Ley *et al*[11]	CN-AML/M1	*FLT3 (27.6), NPM1 (23.9)*	Indels
	
	Mardis *et al*[12]	CN-AML	*NPM1 (23.9), NRAS (9.3), IDH1 (8.5)*	Frame shift insertion, missense
	
	Ley *et al*[13]	CN-AML	*DNMT3A (22.1)*	non-synonymous SNV
	
	Ding *et al*[17]	CN-AML	*TTN, DNMT3A, NPM1, FLT3, WT1, RUNX1, IDH2*	non-synonymous SNVs

Whole Genome, Exome seq	Cancer Genome Atlas Research Network [88]	De-novo AML	*NPM1, FLT3, DNMT3A, IDH1, IDH2, NRAS, RUNX1, TET2*	non-synonymous SNVs

Exome Seq	Yan *et al*[28]	AML-M5	*DNMT3A (20.5), GATA2 (3.6), MLL(19.6)*	Missense, translocation
	
	Grossmann *et al*[30]	CN-AML (NPM^-^FLT^-^CEBPA^-^MLL^-^)	*BCOR (17.1)*	disruptive mutation
	
	Grief *et al*[31]	BiCEBPA^+^ AML	*GATA2 (39.4)*	Missense mutation
	
	Opatz *et al*[32]	CBF leukemia	*FLT3-N676K (6)*	Missense mutation

Targeted DNA capture	Conte *et al*[34]	CN-AML	*FLT3, NPM1, CEBPA, DNMT3A, TET2, IDH1, IDH2, WT1, RAS*	Single base substitution, indels

Transcriptome Seq	Grief *et al*[29]	AML-M1	TLE4, SHKBP1, RUNX1	Missense mutation, Stop mutation
	
	Wen *et al*[22]	CN-AML	*CIITA-DEXI fusion transcript (14/29)*	
	
	Masetti *et al*[23]	pediatric CN-AML	*CBFA2T3-GLIS2 fusion transcript (8.4)*	
	
	Masetti *et al*[24]	pediatric CN-AML CBFA2T3-GLIS2 positive	*DHH-RHEBL1 fusion transcript ( 40)*	

Abbreviations: CN-AML: cytogenetically normal AML; CBF: core binding factor; SNV: single nucleotide variant; CN-AML (NPM^-^FLT^-^CEBPA^-^MLL^-^): CN-AML without mutation in NPM1, FLT3, CEBPA and MLL; BiCEBPA^+^: biallelic CEBPAs

**Table 2:** Candidate gene resequencing studies of *DNMT3A, IDH1, IDH2, TET2 *and *ASXL1* in acute myeloid leukemia

Gene	Reference Study	AML Group	Mutation frequency	Clinical characteristics	Cytogenetics	Association with other mutations	Outcome
***DNMT3A***	Roller *et al*[50]	194 CN-AML	36.10%	Associated with female gender and younger age	All cases are CN-AML	*NPM1*, *FLT3-ITD* and *IDH1*.	NO effect on OS
	
	Ostronoff *et al*[48]	191 selected AML	19%	Significant association with age, gender, WBC count	75% DNMT3Amut cases are CN-AML	*NPM1*	In CN-AML cases, DNMT3A mut has worse OS, EFS and RFS
	
	Ribeiro *et al*[49]	415 AML	23.10%	Associated with higher age, higher WBC and platelet counts	Significant in CN-AML	*FLT3-ITD*, *NPM1* and *IDH1.*	No effect on CR. DNMT3A mut cases have worse OS and RFS
	
	Marcucci *et al*[89]	415 CN-AML	34.20%	Associated with higher WBC count, BM blast percentage	All cases are CN-AML	*NPM1*, *FLT3-ITD*	No effect on CR, DNMT3A mut associated with shorter DFS.
	
	Renneville *et al*[90]	123 younger CN-AML	29.30%	No significant association with age, sex and WBC count	FAB M4/M5	*NPM1*. Inverse association with *CEBPA.*	Lower CR, shorter EFS and OS. DNMT3A mut with NPM1 have inferior EFS and OS
	
	Thol *et al*[89]	489 AML younger than 60yrs	17.80%	Associated with old age, high WBC and platelet counts	Significantly associated with normal karyotype	*NPM1*, *FLT3* and *IDH1*	Shorter OS. In CN-AML shorter OS and lower CR rate

***IDH***	Yamaguchi *et al*[91]	233 Adult AML	8.6% (*IDH1*), 8.2% (*IDH2*)	Associated with older age, high platelet counts and blast percentage	59% of *IDH* mut cases have normal karyotype	*NPM1*. Not a single *IDH* mut case has *CEBPA* mutation	Low CR rate and no difference in RFS
	
	Koszarska *et al*[37]	376 AML	8.5% (*IDH1*), 7.5% (*IDH2*)	Associated with older age, high platelet counts	Associated with intermediate karyotype	*NPM1*	No significant difference in OS, remission and relapse rates
	
	Patel *et al*[38]	199 AML	6.0% (IDH1), 2.0% (*IDH2*)	No significant association with age, gender and WBC count	Strongly associated with normal cytogenetics	No significant association	No analysis
	
	Nomdedéu *et al*[63]	275 AML	13.1% (*IDH1*), 10.2% (*IDH2*)	No significant association with age, gender and WBC count	45.2% *IDH* mut cases have normal karyotype.73.4% *IDH* mut belongs to FAB M4/5	No association	No difference in survival and relapse. CN-AML with IDH mut have adverse OS and DFS
	
	Chotirat *et al*[92]	230 AML	8.7% (IDH1), 10.4% (*IDH2*)	Associated with older age, high platelet counts	55% *IDH* mut cases have normal karyotype, FAB M2	*NPM1*	No effect
	
	Chao *et al*[93]	195 AML	4.6% (*IDH1*), 11.28% (*IDH2*)	Associated with age but not significant in gender and WBC count	Associated with normal cytogenetics. 68%-FAB M5	*NPM1*	No analysis

***TET2***	Grossmann *et al*[94]	95 CEBPA dm AML	34%	ASXL1, TET2 and DNMT3A mutations are associated with older age and FLT3 associated with younger age		*GATA2*, *ASXL1*, *DNMT3A*	Shorter OS and EFS. Additional mutation with TET2 put worse OS
	
	Weissmann *et al*[95]	318 AML patients	27.40%	Associated with older age and high WBC count	75% of *TET2* mut cases are normal karyotype	Inversely associated with *IDH*	Inferior OS and significant shorter EFS.
	
	Chou *et al*[73]	486 pAML	13.20%	Significant with older age, high WBC count and blast percentage	Significantly associated with normal karyotype	*NPM1* and *ASXL1* are less associated with *TET2* mutation. *IDH1* is mutually exclusive	Shorter OS in CN-AML. No difference in CR rate and relapse-free survival
	
	Metzeler *et al*[72]	427 CN-AML	23%	Associated with older age, high WBC count	ALL cases are CN	*IDH* mutations less frequent with *TET2* mutations, *CEBPA* is more frequent with *TET2*	Shorter EFS and DFS, low CR and shorter OS
	
	Kosmider *et al*[96]	247sAML	19.80%	Associated with male gender, old age and platelet counts	51% *TET2* mut cases are normal karyotype.	No significant association	No effect on OS

***ASXL1***	Pratcorona *et al*[97]	886 AML (775 denovo AML, 24 MDS, 37 tAML)	5.30%	Associated with old age and low WBC count	Associated with FAB M0 type, inversely related to M4 type	Inversely related to *NPM1* and *FLT3-ITD*	Independent poor risk factor for OS
	
	Chou *et al*[40]	501 denovo AML	10.80%	Associated with old age and male sex	Associated with FAB M0 type and isolated trisomy 8	*RUNX1*. Inversely related to *NPM1* and *FLT3-ITD*	Shorter OS
	
	Schnittger *et al*[80]	740 AML with intermediate risk karyotype	17.20%	Associated with old age and low WBC count and male gender	Significantly associated with trisomy 8	*RUNX1*.Inversely correlated with NPM1 and *FLT3-ITD*	Shorter OS and EFS
	
	Metzeler *et al*[41]	423 primary CN-AML	10.40%	Associated with old age and male sex, low WBC and Blast percentage	ELN category of CN-AML: Favorable(*ASXL1* mutation in old patients)	Inversly related to *NPM1* and *FLT3-ITD*	Shorter OS

Abbreviations: AML: Acute Myeloid Leukemia; CN-AML: cytogenetically normal acute myeloid leukemia; pAML: primary AML; sAML: secondary AML; tAML: therapy related AML; WBC: White blood cell; FAB: French American British; CR: complete remission; OS: overall survival; EFS: Event-free survival; DFS: Disease-free survival; RFS: relapse free survival; mut: mutant; dm: double mutant

DNMT3A belongs to a family of DNA methylation enzymes with three known mammalian members namely: DNMT1, DNMT3B and DNMT3A. While DNMT1 is responsible for maintaining DNA methylation patterns through cell division, DNMT3A and DNMT3B are known for de-novo methylation [46]. *DNMT3A* mutation is frequent in CN-AML (29%-36%) [47-52]. The most common mutation type is a missense mutation affecting amino acid R882. AML patients showing *DNMT3A* mutation had worse OS and RFS [49]. *DNMT3A* mutations are associated with higher age, high WBC count and female gender [50]. Marcucci G *et al* showed differential prognostic impact of different types of *DNMT3A* mutations in older vs younger patients: R882 mutations are associated with adverse prognosis in older patients whereas non–R882 mutations are associated with adverse prognosis in younger CN-AML [47]. Recent studies have demonstrated that AML cells having R882H mutation show severely reduced de novo methyltransferase activity and focal hypomethylation at specific CpGs throughout their genomes. This activity has been attributed to the dominant-negative inhibition of wild type DNMT3A by R882H DNMT3A mutant that disrupts DNMT3A tetramerization [53]. A similar finding was reported earlier from a murine embryonic stem cell study that showed: Dnmt3a R878H (corresponding to human R882H) mutant protein inhibits wild-type Dnmt3a/Dnmt3b, suggesting dominant-negative effects of mutant DNMT3a [54]. Another noteworthy fact about *DNMT3A* mutation is its stability in the clonal evolution of AML. In NPM1-mutated AML, Kronke *et al*, demonstrated the loss of NPM1 mutation and the persistence on *DNMT3A* mutation at relapse, suggesting *DNMT3A* mutation as a founder event in AML pathogenesis [55]. Other studies have suggested that the stability of DNMT3A mutations during disease progression could be useful for monitoring residual disease [56]. Using conditional ablation, Challen *et al* have demonstrated that *Dnmt3a* loss in mice impairs long-term HSC differentiation, upregulates multipotency genes and downregulates differentiation factors in HSC [57]. Similarly, the role of inactivating *DNMT3A* mutations in AML might be a key event that shuts down differentiation of long-term HSC, leaving the possibility of a secondary oncogenic event to drive transformation into AML. A more recent study demonstrates that DNMT3A mutations precede NPM1-mutation and are found in the HSC and progenitors at diagnosis and remission. Furthermore, this study also demonstrates that pre-leukemic HSCs bearing DNMT3A mutation generate multilineage engraftment and have a competitive advantage in the immunodeficient mice-based repopulation assays [58]. These recent studies not only suggest DNMT3A mutation as a potential therapeutic target and a marker for tracking residual disease, but also as a marker for pre-leukemic clones [56,58].

Another epigenetic modifier mutated in AML is isocitrate dehydrogenase (IDH) enzyme. IDH family has three different isoforms: IDH1, IDH2 and IDH3 which take part in the metabolic Krebs cycle. IDH1 is localized in the cytoplasm. IDH2 and 3 are present in mitochondria, and these three enzymes catalyze the oxidative decarboxylation of isocitrate to alpha ketoglutarate (α-KG) [59]. *IDH1* mutation was recurrently found in 16% of CN-AML [12]. *IDH2* mutation was also reported in AML by candidate gene sequencing study [60]. Recurrent mutations were found in codon R132 in the case of *IDH1* and at codon R140 or R172 in case of *IDH2*, resulting in gain of function of these enzymes which reduce α-KG to oncometabolite 2-hydroxyglutarate (2-HG) [61]. 2-HG was found to be elevated in the serum of AML patients carrying IDH mutations, and the level of this oncometabolite may be useful as a diagnostic and prognostic indicator [62]. The prognostic impact of *IDH* mutations in AML has been investigated by several studies. In CN-AML patients, *IDH* mutations were associated with adverse OS and disease free survival (DFS). In CN-AML with a favorable genotype (*NPM* or *CEBPA* mutated/*FLT3* wt), *IDH* mutations were associated with poor outcome [60,63-65], thereby indicating a possibility of further refinement of this subgroup of CN-AML. In addition to their diagnostic and prognostic values, IDH mutations have been recognized as important therapeutic targets. Recently, Wang *et al* used AGI-6780, a novel and selective inhibitor of mutant IDH2 to induce differentiation in TF-1 (an erythroleukemia cell line) and primary AML cells [66]. In another study, an inhibitor of mutant IDH1, AGI-5198, has been shown to delay growth and induce differentiation of glioma cells [67], providing a proof-of-concept that IDH mutants can be therapeutically targeted. Phase I clinical trials are ongoing with two orally available, selective, potent inhibitors IDH: AG-221 (for IDH2) and AG-120 (for IDH1) [68,69].

Mutation in the Ten-Eleven Translocation 2 (*TET2*) gene was found in 24% of AML and also in other myeloid cancers [70]. TET2 is the member of TET family of dioxygenases that convert 5-methyl-cytosine (5-mC) to 5-hydroxymethyl-cytosine (5-hmC) [71]. 5-hmc plays an important role in DNA demethylation. *TET2* mutations were found to be significant with older age, high WBC and associated with other genetic alterations like *IDH1* mutations in CN-AML. Patients with *TET2* mutations have shorter OS and lower CR rate [72]. Chou *et al* using mutational screening of primary AML patients demonstrated *TET2* mutation as an unfavorable prognostic factor in AML with intermediate cytogenetics [73]. A study with younger adult AML patients with *TET2* mutations in 7.6% cases found no influence of these mutations on the response to therapy and survival. In addition, *TET2* mutations were found to be mutually exclusive with *IDH* mutations [74]. Comparing de novo and relapse stages of AML, Wakita *et al* demonstrated that mutations in the epigenetic modification genes (*DNMT3A*, *TET2* and *IDH*) are stably present at diagnosis and relapse suggesting them as potential biomarker in minimal residual disease (MRD) monitoring [75].

Mutations in genes that effect posttranslational modification of histones have been well known in AML through MLL1 gene aberrations that include translocations and in-frame duplications [76]. Mutations in ASXL1, a member of Polycomb group of proteins have been observed frequently in myelodysplastic syndromes, chronic myelomonocytic leukemia and AML [77-79]. Schnittger *et al* recently observed that *ASXL1* mutations are more frequent in intermediate risk aberrant karyotype AML (31%) than in CN-AML (12.5%). Patients with *ASXL1* mutations had shorter OS and EFS. In addition, *ASXL1* mutation appeared as an independent adverse factor for OS in multivariate analysis [80]. An earlier study by Metzeler *et al* demonstrated that *ASXL1*-mutated older AML, particularly those within ELN Favorable group have adverse clinical outcomes [81]. Although the biological function of ASXL1 is not fully understood, a number of physical interactions with several proteins have been uncovered. A recent study by Abdel-Wahab *et al* showed that hematopoietic-specific deletion of *Asxl1* resulted in an increase in the number of hematopoietic stem and progenitor cells (HSPC) and caused a progressive myelodysplasia that was transplantable. It was shown that *Asxl1* loss resulted in a global reduction of H3K27 trimethylation and aberrant expression of known hematopoietic regulators [82], raising the possibility of using H3K27 demethylase inhibitors in this situation.

## Conclusion and future directions

Recent high throughput NGS approaches have uncovered a number of novel genetic alterations. The main challenge is to integrate this knowledge into clinical management of AML. As highlighted in the sections above, a number of novel molecular mutations have been validated in studies with large number of AML patients and subsequently being used in prognostic subclassification of AML. *DNMT3A*, *TP53* and *MLL-PTD* mutations, for example have been recently suggested to refine the ELN classification of AML [35]. Likewise, *TET2* and *ASXL1* have been suggested as candidate markers for improvement of CN-AML classification in ELN scheme [72,81]. Prospective and large-scale studies are needed to clarify the influence of many of these novel molecular aberrations in conjunction with well characterized prognosticators (*NPM1*, *FLT3-ITD* and *CEBPA*), on prognosis of AML.

The application of NGS in clinical settings is limited by certain challenges such as: technical difficulties in capture of GC-imbalanced targets, inaccurate reading of repetitive genomic regions and time-consuming data analysis that need special bioinformatics skills. In addition, there is a lack of uniform practice for quality assessment of NGS data. A collaborative approach among scientists, bioinformaticians and physicians coupled with creation of easily accessible and understandable database will greatly enhance and ease the clinical application of NGS.

The NGS based AML sequencing has provided several biological insights into the pathogenesis of leukemia. One key lesson derived from these studies, owing to the simultaneous detection of the whole plethora of mutations in cells, is the cancer genome evolution. Paired analysis of diagnosis and relapse samples have revealed marked changes in the genetic makeup of the cells between these points [17]. It is now understood that in AML multiple subpopulations of genetically heterogeneous cells co-exist. Competition among the subpopulations that harbor driver events thus drives the evolution of cancer either in a linear trajectory, a branched trajectory, or a combination of both, eventually shaping the final tumor composition [18,19]. A better understanding of the AML genome evolution is needed. This will help in understanding the mechanisms of relapse, provide information about driver and cooperating events, improve the understanding of recurrent patterns of therapy resistance and suggest appropriate molecular targets.

One way to understand the pathogenic relevance of novel genetic alterations is to perform functional studies in animal models. A number of studies have taken this approach recently in evaluating the role of: Asxl1 [82], Tet2 [83] and Dnmt3a [57]. However, many recently identified genetic alterations, including novel chromosomal translocations remain to be studied in detail. Exhaustive functional studies using *in vitro* and *in vivo* experimental models are needed to understand the biological significance of these novel alterations and to distinguish driver events from passenger events. In addition, these models would pave the way for preclinical studies including testing novel therapeutic interventions.

Identification of high frequency of mutations in potential epigenetic regulators (*DNMT3A*, *TET2*, *IDH1*, *IDH2*, *ASXL1*, and *UTX*) is particularly interesting. It remains imperative that the mechanisms underlying epigenetic dysregulation and its consequences be clearly understood. This may allow us to better use the available methylation and histone deacetylase (HDAC) inhibitors based on the underlying genetic abnormalities that have shown some clinical success in the treatment of myeloid leukemias [77,84]. Many new drugs that target aberrant but reversible epigenetic modifications have been recently tested in preclinical leukemia models and leukemia cell lines: Schenk *et al* recently demonstrated that inhibitors of lysine-specific demethylase 1 (LSD1, also known as KDM1A) epigenetically reprogram the cells to make them sensitive to ATRA induced differentiation in AML [85]. A study by Deshpande *et al* demonstrated how inhibition of histone-methyltransferase DOT1L, that is the driving force behind MLL-AF6 fusion leukemias, resulted in inhibition of MLL-AF6-transformed cells [86]. Stewart *et al* demonstrated how targeting bromodomain containing protein 4 (BRD4) in combination with daunorubicin inhibited growth of DNMT3A/NPM1-mutated leukemia cells [87]. These studies stress the potential of targeted therapies against the expanding spectrum of mutations in AML.

The progressive enhancement in the molecular characterization of AML holds promise for undertaking the more precise and targeted therapeutic strategies leading to possibly better treatment outcomes. In addition to this, inclusion of NGS based deep sequencing methods in molecular diagnostics workup has the potential to realize the goals of personalized medicine.

## Abbreviations

AML: Acute myeloid leukemia; NGS: next generation sequencing; OS: overall survival; APL: Acute promyelocytic leukemia; CBF: core binding factor; CN-AML: cytogenetically normal acute myeloid leukemia; WGS: Whole genome sequencing; DNMT3A: DNA methyltransferase-3-alpha gene; FISH: Fluorescence in situ hybridization; TCGA: the Cancer Genome Atlas; ncRNA: non-coding RNA; SNP: single nucleotide polymorphism; RNA-seq: RNA Sequencing; biCEBPA: biallelic CEBPA mutation; ZF1 domain: zinc finger domain; FLT3: fms-related tyrosine kinase 3; CBF: core binding factor; SNVs: single nucleotide variants; ELN: European Leukemia Net; CK-AML: complex karyotype; MK: monosomal karyotype; RFS: relapse-free survival; DFS: disease free survival; CR: complete remission; OS: overall survival; EFS: event-free survival; IDH: isocitrate dehydrogenase; α-KG: alpha ketoglutarate; 2-HG: 2-hydroxyglutarate; TET2: Ten-Eleven Translocation 2; 5-mC: 5-methyl-cytosine; 5-hmC: 5-hydroxymethyl-cytosine; MRD: minimal residual disease; HSPC: hematopoietic stem and progenitor cells; HDAC: histone deacetylase

## Competing interests

The authors declare that they have no competing interests.

## Authors’ contributions

FA, AMI and SA wrote the manuscript. MF, MIN, TAK, MHA and MG edited the final version.

All authors read and approved the final version of MS.

## Declarations

Publication charges for this article have been funded by the Center of Excellence in Genomic Medicine Research, King Abdulaziz University, Jeddah.

This article has been published as part of *BMC Genomics* Volume 16 Supplement 1, 2015: Selected articles from the 2nd International Genomic Medical Conference (IGMC 2013): Genomics. The full contents of the supplement are available online at http://www.biomedcentral.com/bmcgenomics/supplements/16/S1
